# Long-Acting Injectable Cabotegravir for HIV Prevention: What Do We Know and Need to Know about the Risks and Consequences of Cabotegravir Resistance?

**DOI:** 10.1007/s11904-022-00616-y

**Published:** 2022-09-16

**Authors:** Urvi M. Parikh, Catherine A. Koss, John W. Mellors

**Affiliations:** 1 Division of Infectious Diseases, University of Pittsburgh School of Medicine, Pittsburgh, USA; 2 Division of HIV, Infectious Diseases, and Global Medicine, Department of Medicine, University of California, San Francisco, San Francisco, USA

**Keywords:** HIV prevention, Pre-exposure prophylaxis, Cabotegravir, HIV drug resistance, Integrase strand transfer inhibitors (INSTI)

## Abstract

**Purpose of Review:**

Cabotegravir is a potent integrase strand transfer inhibitor (INSTI) recently approved as a long-acting injectable formulation for HIV prevention (CAB-LA). We summarize what is known about cabotegravir pharmacokinetics, activity, and emergence of resistance from in vitro, macaque and clinical studies, and we evaluate the risk of resistance from CAB-LA with on-time injections and after CAB-LA discontinuation.

**Recent Findings:**

The accumulation of multiple INSTI mutations is required for high-level cabotegravir resistance, and the same mutation combinations may cause cross-resistance to dolutegravir, which is widely used for first-line antiretroviral therapy in low- and middle-income countries. Though CAB-LA was highly effective in preventing HIV, breakthrough infections did occur in trials of CAB-LA despite on-time injections, resulting in selection of single and combinations of INSTI resistance mutations.

**Summary:**

As CAB-LA is scaled-up, prompt HIV diagnosis to prevent resistance, and resistance monitoring could help preserve the effectiveness of INSTIs for both HIV treatment and prevention.

## Introduction

Antiretroviral (ARV)-based prevention is a critical component of initiatives to end the HIV epidemic in the USA and globally [1]. An estimated 1.5 million people acquired HIV globally in 2020, far exceeding the UNAIDS 2020 target of 500,000 new infections, and highlighting a pressing need for effective prevention strategies to slow the spread of HIV [2]. Oral pre-exposure prophylaxis (PrEP) with a fixed-dose combination of tenofovir disoproxil fumarate and emtricitabine (TDF/FTC) was approved by the U.S. Food and Drug Administration (FDA) in 2012 [3–8] and has since been implemented for daily oral use across more than 75 countries with over 600,000 individuals receiving PrEP in 2019 [9]. Tenofovir alafenamide with emtricitabine (F/TAF), taken orally once a day, was approved by the U.S. FDA in 2019 for non-vaginal exposures [10] and is currently under evaluation for use by ciswomen in Sub-Saharan Africa (SSA) (NCT04994509). The non-nucleoside reverse transcriptase inhibitor (NNRTI) dapivirine, formulated into a silicone elastomer vaginal ring (self-inserted monthly), was recommended by the World Health Organization (WHO) in January 2021 after two phase III trials and open-label extension studies showed a 27–39% reduction in HIV incidence in African women [11–15]. Numerous countries in Africa, including South Africa and Zimbabwe, are in various stages of dapivirine ring approval with their relevant regulatory authorities.

The efficacy for HIV prevention of oral and intravaginal PrEP is highly dependent on the level of product adherence. Potent long-acting antiretroviral agents requiring less frequent dosing may partially overcome adherence challenges and provide longer periods of HIV protection to achieve higher product effectiveness [16]. An extended-release integrase strand transfer inhibitor (INSTI), long-acting cabotegravir (CAB-LA), given every 8 weeks through intramuscular injection, was recently shown to be safe, well-tolerated, and highly efficacious in phase III clinical studies of HIV prevention. CAB-LA demonstrated a 66% reduction in HIV infection among transwomen and men who have sex with men (MSM) at multiple global sites and an 89% reduction among ciswomen in Sub-Saharan Africa relative to TDF/FTC [17••, 18••, 19••, 20••], and showed an even greater estimated efficacy of 93–95% compared to a calculated placebo rate [21]. The FDA approved CAB-LA in December 2021 for use as injectable PrEP and WHO released guidelines for use of CAB-LA as PrEP in 2022 [22].

**Fig. 1:**
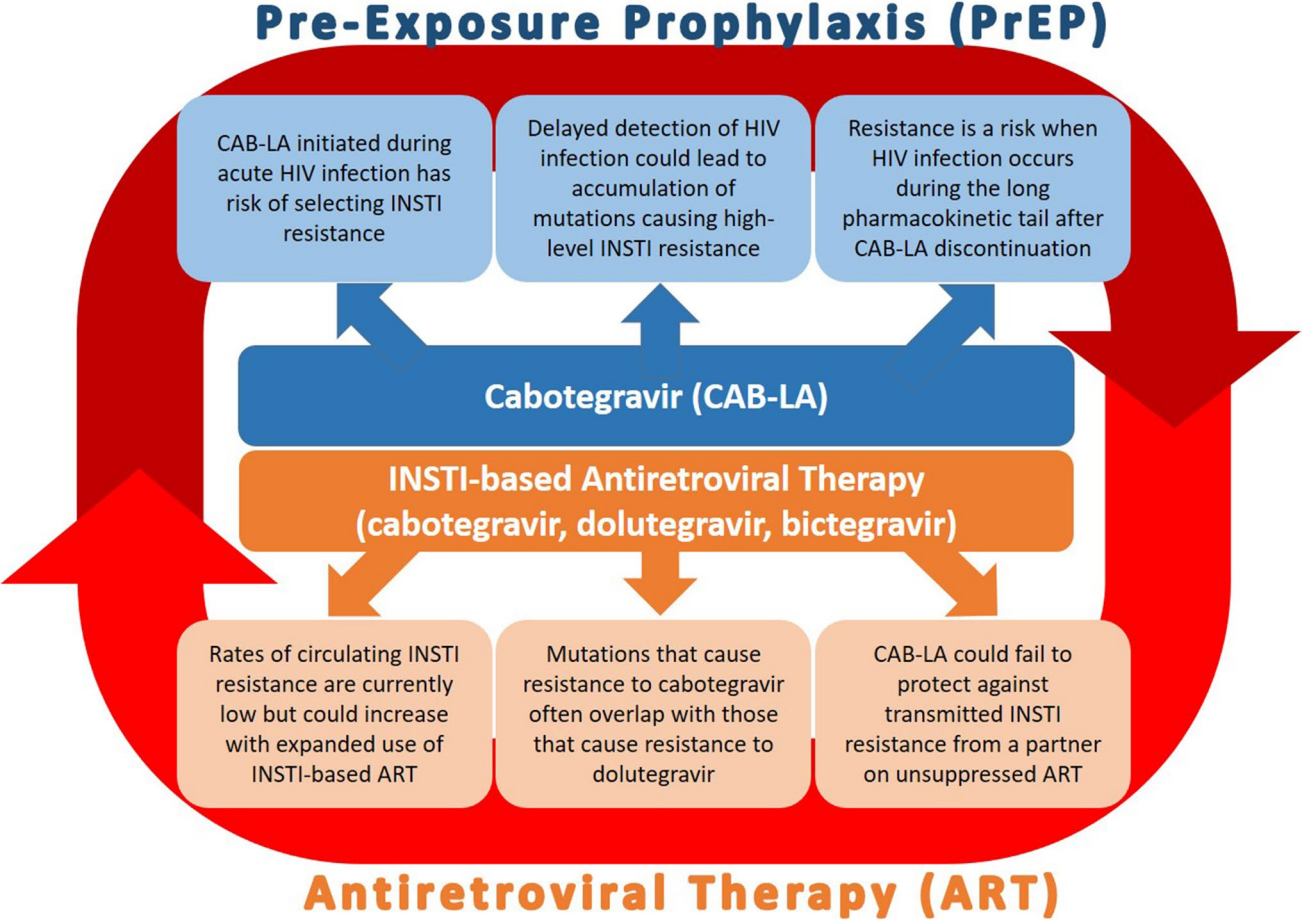
Causes and potential consequences of cabotegravir resistance for HIV prevention and treatment

Despite the tremendous promise of CAB-LA to reduce HIV incidence, there are concerns about the risk of resistance among persons who are diagnosed with HIV after starting CAB-LA and the potential consequences of cabotegravir resistance for HIV treatment with antiretroviral therapy (ART). First-line ART regimens in the USA and globally include INSTIs in the same class as cabotegravir, such as oral dolutegravir (recommended by WHO as first line ART), bictegravir, and a new injectable combination of cabotegravir and rilpivirine (RPV). Using the same drugs and drug classes for both prevention and treatment increases the risk of resistance and failure of both approaches. Starting or continuing to give CAB-LA for prevention after HIV has been acquired but before diagnosis, could select for drug resistance and potentially lead to virologic failure of INSTI-based ART when it is initiated. Moreover, cabotegravir can persist in the body for 1 year or more after the last injection in a long pharmacokinetic (PK) “tail,” increasing the period of risk for breakthrough infection and selection of cabotegravir resistance [23••]. Although the prevalence of INSTI pre-treatment drug resistance, defined by the WHO as HIV drug resistance in individuals starting or re-starting ART, is currently very low (< 1%) in Africa, it could rise as DTG use expands in countries that used fixed-dose first line regimens, thereby reducing the efficacy of CAB because of INSTI resistance transmitted from a partner [24] (Fig. 1).

Here, we examine the risk of cabotegravir resistance and cross-resistance to other INSTIs with the rollout of CAB-LA for HIV prevention, focusing on (i) what is currently known about cabotegravir resistance and cross-resistance from in vitro, macaque model and human studies; (ii) what is currently known about cabotegravir pharmacokinetics with long-acting injectable use; and (iii) the implications of these findings for HIV prevention and treatment.

## Cabotegravir Resistance Observed In Vitro and in Clinical Studies

### In Vitro Studies Have Identified Mutation Combinations That Reduce Susceptibility to Cabotegravir

Cabotegravir (GSK1265744 or GSK744) was discovered in 2013 as a modification of dolutegravir; both are carbomoyl pyridone analogues, with cabotegravir containing chemical features to enable long-acting monthly or less frequent dosing and structural elements that prevent rapid selection of resistance [25, 26]. Unlike nucleoside/nucleotide reverse transcriptase inhibitors (NRTIs) and NNRTIs, single coding mutations are generally insufficient to reduce the potency of cabotegravir. Studies of systemically generated site-directed mutants tested in single cycle indicator cell assays have identified several key mutation combinations that confer moderate (> tenfold) to high level (> 100-fold) resistance to cabotegravir. These mutations include amino acid changes at positions 74, 97, 138, 140, 147, 148, 155, and 263 in the *integrase* gene. Combinations of two to four of these mutations confer > tenfold resistance, with the highest level of resistance (> 1000-fold) observed with T97A/G140S/G148H with or without E138A. Many, but not all, of these mutations track closely with the resistance profile of dolutegravir. A large panel of clinical isolates was tested against cabotegravir to ensure that HIV integrase polymorphisms in different subtypes are not naturally resistant to cabotegravir (Table 1) [26–28, 29•, 30•, 31–34, 35•]. Mutations in Table 1 were classified as major, polymorphic, or accessory dependent upon their inclusion in the 2019 IAS-USA list and/or Stanford Drug Resistance database [36–38].

**Table 1:** Major integrase gene resistance mutation and mutation combinations associated with cabotegravir resistance or cross-resistance based on in vitro studies

Category	Major mutations or mutation combination in the HIV-1 *integrase* gene^a^	Polymorphic and/or accessory INSTI mutations^b^	Level of resistance or cross-resistance conferred
Single Mutants	G118R, Q148KR, R263K	None	Low (5–tenfold)
Double Mutants	Combinations of two mutations at positions L74M, T66K, E138AK, G140ACS, Q148HKR, N155H and/or R263K	Any major mutation + E92Q, Y143H or S147G	Moderate to High (> tenfold)
Triple Mutants	G140ACS + Q148RHK + a third mutation, including L74M or E138AK	Combinations of two major mutations + T97A, Y143R, G149A, T122N, G147S or G163K	Moderate to High (> tenfold)
Quadruple Mutants	Four mutations in any combination of the following variants: L74M, E138AK, G140AS, Q148AHKR, N155H and/or R263K	Combinations of three major mutations + T97A C56S, S147G Q148A G149A or V75A	High (> 100-fold)

^a^Mutations were identified through multiple published studies that used one or more of the following approaches: (i) laboratory generated molecular clones in cell-based reporter assays; (ii) recombinant virus from patient plasma in MT4 phenotyping assay; (iii) cell-culture selections against primary isolates from newly infected individuals; and (iv) phenotyping studies of INSTI-experienced patients on failing antiretroviral therapy. Mutations in this list are included in the 2019 IAS-USA drug resistance update and/or listed in the Stanford resistance database as major INSTI mutations with the exception of L74M, which is not on the 2019 IAS-USA list, but conferred moderate to high-level resistance in combination with other INSTI mutations

^b^Mutations in this list are not included in the 2019 IAS-USA drug resistance update and/or are listed in the Stanford resistance database as polymorphic, accessory, or other mutations. Some mutations in this list may be major for INSTIs that are not typically cross-resistance with cabotegravir such as elvitegravir and raltegravir

### High Level Resistance Is Selected in Macaques Administered Cabotegravir as Pre-Exposure Prophylaxis

The first studies in rhesus macaques investigated single-dose and monthly CAB-LA with weekly SHIV162P3 intrarectal challenges. Among 8 male macaques, there were three breakthrough infections, but no INSTI resistance [39]. To specifically evaluate the risk of resistance with starting CAB-LA during acute HIV infection, two injections of CAB-LA, 1 month apart, were administered to rhesus macaques 11 days after intravenous infection with RT-SHIV. Three of six macaques developed high-level cabotegravir resistance with the mutations G140R, E92Q/G140R, G118R, G118R/A122T (> 1000-fold). Of these mutations, G118R and A122T were newly identified as mutations associated with CAB [40•].

### What Did We Learn About CAB Resistance from HIV Treatment Trials?

The LATTE, LATTE-2, FLAIR, ATLAS, and ATLAS-2 M trials evaluated the combination of cabotegravir with rilpivirine (CAB/RPV) as an oral pill or monthly injection for treatment of persons living with HIV. Evaluation of resistance in individuals who did not achieve virologic suppression on CAB/RPV can provide insight into the mutational patterns selected in vivo and confirm the clinical importance of mutations identified through in vitro studies (Table 1). Altogether, in the five studies, 15 of 25 individuals with breakthrough viremia (defined as HIV-1 RNA ≥ 50 copies/ml at the study endpoint) had cabotegravir resistance, with mutation patterns that included Q148R with other INSTI mutations including T97A, G140R, E138K, Q148R, N155H, and R263L [41, 42•, 43, 44•] (Table 2). As noted in the in vitro studies, multiple mutations in combination were necessary to confer cabotegravir resistance in viremic patients.

**Table 2:** Cumulative summary of integrase gene mutations observed in participants with breakthrough viremia or infection in treatment and prevention trials of Cabotegravir

Number with Cabotegravir resistance	Trials	Mutations observed
15 of 25 (60%)	LATTE, LATTE-2, FLAIR, ATLAS, ATLAS-2 M	L74I, T97A, G140R, E138K, Q148ER, N155H, R263L
7 of 20 (35%)^a^	HPTN-083, HPTN-084	L74I, E138ADK, G140AS, Q146LR, Q148R, N155H, E157Q, S230R, R263K

^a^Resistance data is available for 16 of 35 infections in the CAB-LA arm of HPTN-083, and 4 infections in the CAB-LA arm of HPTN-084

Polymorphisms at codon 74 in *integrase* are of specific interest. In combination with other INSTI mutations, L74M caused moderate to high-level resistance to cabotegravir in vitro (Table 1). In participants from the FLAIR study with L74I at baseline who subsequently experienced virologic failure on cabotegravir-based ART, reduced susceptibility to cabotegravir was only observed when L74I occurred in combination with Q148R. L74I/G140R mutants remained susceptible to cabotegravir and time to breakthrough viremia was similar in participants with and without L74I at baseline [45•]. L74I is proposed to restore replication capacity when present in combination with other INSTI mutations [46]. Early detection of INSTI resistance could prevent accumulation of mutations that confer higher levels of resistance to cabotegravir.

## Resistance Risk in Trials of Long-Acting Cabotegravir for HIV Prevention

To date, two randomized phase III double-blind, placebo-controlled trials have evaluated the efficacy of CAB-LA in preventing HIV infection in 4566 cisgender men and transgender women who have sex with men (HPTN-083) from 43 sites in Argentina, Brazil, Peru, USA, South Africa, Thailand, and Vietnam, and, in 3224 cisgender women of reproductive age (18–45 years of age) from 20 sites in Uganda, Kenya, Malawi, Zimbabwe, Eswatini, South Africa, and Botswana [17••, 18••]. The CAB-LA arm of both studies had a 5-week lead-in phase with daily oral cabotegravir (30 mg) followed by 600 mg gluteal intramuscular injection of CAB-LA given at week 5, 9, and every 8 weeks (as well as daily oral placebo tablets) for approximately 3 years. Participants who stopped CAB-LA injections were offered open-label daily oral TDF/FTC for 48 weeks to cover the cabotegravir PK tail. Individuals randomized to the TDF-FTC arm received daily oral tablets and placebo injections at weeks 5, 9, and every 8 weeks. The oral lead-in ensured that there were no safety concerns prior to administration of the first long-acting injection if acute HIV infection was retrospectively detected by plasma HIV-1 RNA testing on the sample collected at study entry. The extensive tail coverage with TDF-FTC was included to mitigate the risk of resistance during waning concentrations of CAB that could persist for > 1 year after the last injection, resulting in sub-therapeutic drug concentrations that are too low to protect against HIV-1 infection but provide enough selective drug pressure for CAB resistance to emerge [23••, 47, 48].

### Infections at Baseline and After Initiating Long-Acting Cabotegravir

In HPTN-083, a total of 35 HIV infections occurred in the CAB-LA arm. Of the 25 incident HIV infections in the CAB-LA arm from the primary blinded and 1 year unblinded analysis period, 3 occurred during oral lead-in period, 12 occurred after lapses in injections (more than 6 months after last CAB-LA exposure), 3 in individuals who mostly had on time injections but with one or more injection that was delayed by > 8 weeks, and 7 occurred despite on-time injections. An additional 10 infections that were not included in the incidence analysis was comprised of four individuals who started CAB-LA with undetected acute HIV infection, and six individuals who seroconverted during the planned 1 year TDF/FTC oral PrEP extension after discontinuing CAB-LA. Of these 35 infections, data on HIV drug resistance have been reported for 16 participants to date [17••, 18••, 49].

Seven of 16 (44%) infections in the CAB-LA arm after cabotegravir exposure developed INSTI resistance. Each case had a unique pathway to resistance, starting with a known INSTI mutation with gradual accumulation of additional mutations yielding high-level cabotegravir resistance. Mutations identified were similar to those reported in vitro and from CAB-LA-treated individuals with virologic failure, and included the amino acid changes L74I, E138K, Q148R, EE157Q, N155H, and R263K among others [17••, 18••, 49] (Table 2).

These findings highlight important concerns. First, detection of infection was delayed due to suppressed viral loads from cabotegravir, leading to a prolonged time to seroconversion as detected by antibody or antigen/antibody testing (ranging from 45 to 117 days after first positive RNA, as determined retrospectively). Continued viral evolution during ongoing cabotegravir exposure resulted in the accumulation of multiple INSTI mutations that together cause higher level resistance than single mutations. Thus, earlier detection of infection would have enabled earlier ART initiation, potentially preventing the development of high-level INSTI resistance. Second, 5 of the 6 resistant cases occurred despite on-time injections. The reason on-time injections did not confer a sufficiently high enough concentration of CAB-LA to prevent infection and resistance is not yet known [17••, 18••, 49].

In HPTN-084, Long-Acting Injectable for the Epidemic (LIFE) study, which evaluated the efficacy of CAB-LA in women in Sub-Saharan Africa, no INSTI mutations were detected in the 4 seroconversions observed (1 baseline infection and 3 incident infections) in the CAB-LA arm [20••].

## PK Studies: What Do We Know About the Tail?

The tail phase of CAB-LA was assessed among participants in the Study to Evaluate the Safety Tolerability and Acceptability of Long Acting Injections of the Human Immunodeficiency Virus (HIV) Integrase Inhibitor, GSK1265744, in HIV Uninfected Men (ECLAIR), a phase 2 randomized, placebo-controlled trial that enrolled HIV-uninfected male volunteers and provided CAB-LA 800 mg injections every 12 weeks. Overall, 17% of participants had detectable plasma cabotegravir concentrations 52 weeks after the last injection. Notably, because the dose of CAB-LA used in ECLAIR did not meet PK targets derived from non-human primate studies, a different dosing regimen was studied in the phase 3 CAB-LA PrEP trials (600 mg given 4 weeks apart, followed by 600 mg every 8 weeks) [48].

Tail phase PK was also assessed among healthy HIV-uninfected men and women in the HPTN 077 trial. HPTN 077 was a double-blind, randomized, placebo-controlled phase IIa trial conducted at 8 global sites that included 600 mg and 800 mg dosing regimens. Median time from last CAB-LA injection to cabotegravir concentrations decreasing below the lower-limit of quantitation (LLOQ) was 43.7 weeks (IQR 31.1–66.6; range 20.4–152.5) for male participants compared to 67.3 weeks (IQR 29.1–89.6; range 17.7–225.5) for female participants. The time to LLOQ was approximately 30% longer among women than men. Moreover, the apparent terminal-phase half-life of CAB was approximately 31% longer among participants with a higher body mass index (BMI). Of note, however, less than 10% of PK variability was explained by sex and BMI. The investigators note that additional factors, such as muscle size, muscle-fat content, host genetics, and potential delivery of injections into subcutaneous tissues or intravenously rather than intramuscularly should also be explored as potential contributors [23••].

### Knowledge Gaps on CAB-LA as PrEP

Despite concerns about the potential for INSTI resistance during the CAB-LA PK tail, little is currently known about the risk of resistance after stopping CAB-LA injections. Incident HIV infections reported from the CAB-LA PrEP trials have not occurred during the PK tail, potentially because these infections occurred during active product use and because the trial protocols included the use of daily oral TDF/FTC for 48 weeks after the last CAB-LA injection to protect against HIV acquisition during waning cabotegravir concentrations. Additional data are anticipated on the risk of resistance during the CAB-LA PK tail with longer follow-up of trial participants during the open-label extensions of HPTN-083 and 084. Moreover, with use of CAB-LA PrEP in routine clinical settings, where injections may be stopped or missed without switching to oral PrEP or oral CAB to cover the PK tail, further data on the risk of resistance during the tail phase may be forthcoming.

### Special Considerations: PK Variability in Different Populations

As CAB-LA PrEP rolls out in routine clinical settings, PK variability in diverse populations could result in higher or lower drug concentrations and influence both protection from HIV acquisition and the risk of ARV resistance. CAB-LA PK during pregnancy is of high interest given that women of childbearing age, particularly in Sub-Saharan Africa, account for a disproportionate burden of new HIV infections globally [50]. Moreover, the risk of HIV acquisition may be elevated during pregnancy and pregnant women are thus a priority population for HIV prevention [51]. Plasma concentrations of several ARVs are altered during pregnancy, particularly during the third trimester [52]. Inadequate exposure to an ARV for prevention during pregnancy could lead to HIV acquisition and perinatal transmission to the infant. Notably, for dolutegravir use for HIV treatment, the area under the curve (AUC) may be decreased during the third trimester of pregnancy, but no dose adjustment has been recommended during pregnancy [52].

Data are currently limited on cabotegravir PK among pregnant persons. Data have been reported among a small number of women who became pregnant while receiving CAB/RPV (oral or LA) for HIV treatment; CAB/RPV was discontinued upon identification of pregnancy and quarterly PK sampling was performed for 52 weeks following the last injection. Plasma cabotegravir concentrations were reported to be within the range of expected concentrations for non-pregnant women [53]. The HPTN 084 CAB-LA PrEP trial required participants to use long-acting contraception, although pregnancies did occur during the phase III trial. After pregnancy was diagnosed, participants were unblinded and no further CAB-LA was received. Participants were offered daily oral TDF/FTC for prevention (similar to non-pregnant participants who discontinued CAB-LA injection). An analysis of participants who received CAB-LA until pregnancy diagnosis in HPTN 084 found that cabotegravir concentrations were similar to those in non-pregnant women in HPTN 077 [23••]. Age, weight, race, and pregnancy status were not associated with changes in the apparent terminal phase half-life (*t*_1/2app_) of CAB-LA. Higher BMI (> 27.2 kg/m^2^) was associated with longer CAB-LA *t*_1/2app_ (fold-change 1.49, *p* = 0.069) [54]. Notably, in the results reported to date, CAB-LA was only given in very early pregnancy, before pregnancy was detected by frequent testing in the trial. Therefore, CAB-LA PK with continued injections during the second and third trimesters remains unknown. The open-label extension (OLE) of HPTN 084 does not require long-acting contraception and participants will be allowed to continue active dosing of CAB-LA during pregnancy and will be followed to assess pregnancy and infant outcomes. Therefore, additional data on CAB-LA PK during pregnancy and the postpartum period, as well as cabotegravir exposure to infants in utero and during breastfeeding, are anticipated from the HPTN 084 OLE and other studies.

Potential drug interactions between CAB-LA and hormone therapy are another important consideration for CAB-LA PrEP use among diverse populations. A study of repeat dosing of cabotegravir 30 mg daily found no significant impact on the PK of oral contraceptives (OC) containing levonorgestrel and ethinyl estradiol, and the FDA label states that no dose adjustments are needed with co-administration [55]. In a secondary analysis of ciswomen in the phase II HPTN 077 trial, the use of oral hormonal contraceptives was associated with significantly lower peak cabotegravir concentrations compared to women not on hormonal contraception [56•]. No differences in other CAB-LA PK parameters were observed, nor were differences seen with injectable, implantable, or other contraceptives. The phase III HPTN 084 CAB-LA PrEP trial required the use of long-acting reversible contraception (LARC) and did not find differences in HIV incidence across LARC methods, although there were only four HIV infections in the CAB-LA arm, thus limiting comparisons.

Transgender individuals are another priority population for HIV prevention, given the higher burden of HIV infection among trans persons in the USA and globally [57]. Several studies have investigated the impact of tenofovir-based PrEP on gender-affirming hormone therapy (GAHT) concentrations among transgender individuals, and the impact of various GAHT regimens on tenofovir-based PrEP [57–59]. In the HPTN 083 phase 3 trial, 12.5% of participants were transwomen who have sex with men. The point estimate for reduction in HIV incidence was similar among transwomen to MSM in the trial, but was not statistically significant due to smaller numbers in this subgroup (HR 0.34, 95% CI 0.08–1.56) [17••]. A preliminary analysis of PK data from HPTN 083 found that plasma concentrations of CAB were similar among transwomen receiving and not receiving GAHT [60].

## Diagnostics to Mitigate Resistance Risk

The FDA label for CAB-LA PrEP includes warnings about the risk of resistance with the use of CAB-LA during acute HIV infection and recommends use of antigen/antibody diagnostic tests prior to initiating or continuing CAB-LA, and confirming negative results with an HIV RNA-specific assay. An oral lead-in is considered optional in the FDA label. The stringent HIV testing recommendations for CAB-LA could pose implementation challenges in sub-Saharan Africa, where fourth-generation antigen/antibody rapid tests are not in use as part of national algorithms, and viral load scale up is ongoing but not routinely available with rapid turnaround time, in all settings.

Several planned studies are aiming to address these significant implementation challenges. The HPTN 083/084 open label extensions and other studies, including the Catalyzing access to new prevention products to stop HIV (CATALYST) study, by the PEPFAR/USAID/FHI 360 Maximizing Options to Advance Informed Choice for HIV Prevention (MOSAIC) consortium, will provide additional data on the use of different HIV testing algorithms, the number of infections and incidence of resistance, and pregnancy and infant outcomes and PK during pregnancy.

## Impact of CAB Resistance on HIV Treatment Outcomes

The success of dolutegravir-based first line ART in individuals who have breakthrough HIV infection with INSTI mutations on CAB-LA PrEP is currently not known and remains an important area of future investigation. Notably, in the HPTN 083/084 trials, individuals who acquired HIV were treated with non-INSTI regimens (e.g., NNRTI or protease inhibitor-based) and thus the impact of CAB-LA resistance on treatment with first-line INSTI-based ART remains unknown. The Nucleosides and Darunavir/Dolutegravir in Africa (NADIA) study demonstrated effectiveness in using dolutegravir with NRTIs to treat patients with NRTI resistance [61], but this finding may not hold up in patients with cabotegravir resistance.

Given the structural similarities of dolutegravir with cabotegravir and overlapping cross-resistance profiles, assessing virologic outcomes of dolutegravir-based ART in individuals who seroconvert on CAB-LA will be important for developing guidance for optimal choice of therapy for those with prior CAB-LA exposure.

## Conclusions

CAB-LA PrEP represents a tremendous advance in the menu of HIV prevention options and holds great promise to reduce HIV incidence. A potential “cost” of rolling out this highly efficacious prevention intervention may be the risk of INSTI resistance among persons who acquire HIV while using CAB-LA PrEP [62]. Further, high-level cabotegravir resistance could reduce the efficacy of dolutegravir and other INSTIs that are used as first-line treatment for HIV across global settings, thereby increasing the risk of virologic failure on first-line ART and the potential for onward transmission of INSTI resistance. As CAB-LA PrEP is scaled up globally, there is thus a pressing need for robust monitoring for incident HIV infections and INSTI resistance among persons using CAB-LA PrEP, as well as levels of background INSTI resistance in communities where CAB-LA is rolled out. Further refinement of HIV testing algorithms to improve HIV diagnosis among persons using CAB-LA and expansion of INSTI resistance testing could minimize potential negative outcomes from rollout of CAB-LA for prevention. Overall, there are substantial HIV prevention benefits of deploying CAB-LA globally. As this highly promising prevention modality is scaled up, more sensitive diagnostic tools and expanded resistance testing may help sustain the effectiveness of INSTIs for both HIV treatment and prevention.
